# An Efficient Intergeneric Conjugation of DNA from *Escherichia coli* to Mycelia of the Lincomycin-Producer *Streptomyces lincolnensis*

**DOI:** 10.3390/ijms13044797

**Published:** 2012-04-16

**Authors:** Lei Du, Rui-Hua Liu, Li Ying, Guang-Rong Zhao

**Affiliations:** 1 Key Laboratory of Systems Bioengineering, Ministry of Education, Department of Pharmaceutical Engineering, School of Chemical Engineering and Technology, Tianjin University, P.O. Box 6888, Tianjin 300072, China; E-Mail: dulei198609@163.com; 2 Anhui Wanbei Pharmaceutical Co., Ltd. No.24, Huaihai Road, Suzhou 234000, China; E-Mails: rh_liu001@126.com (R.-H.L.); elin65521@sina.com (L.Y.)

**Keywords:** *Streptomyces lincolnensis*, lincomycin, intergeneric conjugation, *E. coli*-mycelia conjugation, *lmrABC*

## Abstract

*Streptomyces lincolnensis* is a producer of lincomycin, which is a lincosamide antibiotic for the treatment of infective diseases caused by Gram-positive bacteria. *S. lincolnensis* is refractory to introducing plasmid DNA into cells because of resistance of foreign DNAs and poor sporulation. In this study, a simple and efficient method of transferring plasmids into *S. lincolnensis* through the intergeneric *Escherichia coli*-mycelia conjugation was established and optimized for the first time. The recipient mycelia of *S. lincolnensis* were prepared in liquid SM medium containing 10.3% sucrose for three days. The dispersed mycelia were conjugated with competent *E. coli* donor cells. The exconjugants were regenerated efficiently on solid mannitol soya flour (MS) medium containing 20 mM MgCl_2_. The average conjugation frequency was observed at 1.1 × 10^−4^ per input donor cell and validated functionally by transferring two types of vectors containing lincomycin resistance genes *lmrA*, *lmrB* and *lmrC* into *S. lincolnensis* mycelia. The data of fermentation in shaking flasks showed the lincomycin yield of the exconjugants increased by 52.9% for the multiple copy vector and 38.3% for the integrative one, compared with the parental strain. The efficient and convenient method of intergeneric *E. coli*-mycelia conjugation in this study provides a promising procedure to introduce plasmid DNA into other refractory streptomycetes.

## 1. Introduction

Lincomycin and its 7-chloro-7-deoxy derivative clindamycin are lincosamide antibiotics composed of propylproline and methylthiolincosamide [1]. Lincomycin, produced by *Streptomyces lincolnensis*, is used as a major antibiotic for the treatment of diseases caused by Gram-positive bacteria [1]. Clindamycin is usually used to treat infections caused by anaerobic bacteria and some protozoal diseases, such as malaria, infections of the respiratory tract, skin and soft tissue infections, and peritonitis [2]. It is a common topical treatment for acne [3] and can be useful against some methicillin-resistant *Staphylococcus aureus* infections [2]. Lincomycin biosynthetic gene cluster had been cloned and characterized [4,5]. The importance of fermentative production of lincosamide antibiotics [6] and the lack of efficient techniques to introduce plasmid DNA into *S. lincolnensis,* have encouraged the development of an efficient DNA manipulation technology to improve productivity and to analyze functionality of the secondary metabolite genes of interest in *S. lincolnensis*.

Two genetic transformation systems have been developed based on the model strain *Streptomyces coelicolor* A3(2) [7]. The protoplast transformation is widely used as the most common method for transferring foreign plasmid to streptomycetes. However, the efficiency of the protoplast transformation system varies significantly from one species to another, so it is necessary to improve the experimental procedures for a new species with a strong restriction to foreign DNAs. Few studies have reported methods of transferring DNA into *S. lincolnensis* mainly because of the resistance and modification of foreign DNAs [8]. The intergeneric conjugation between *E. coli* and streptomycetes was first reported by Mazodier [9] and spore conjugation methods have successfully been applied in various *Streptomyces* species [10–13]. For streptomycetes with poor sporulation, spore conjugation was not always successful or had extremely low conjugation frequency. Mycelia are the vegetative and propagation forms of actinobacteria, and have potency in the intergeneric conjugation. Some research results concerning plasmid transferring methods using mycelia have been reported for several *Streptomyces* [14–16], *Streptosporangiaceae* [13] and *Saccharopolyspora spinosa* [17] from *E. coli*. In our early studies, we tried to transfer foreign plasmid into *S. lincolnensis* through the two available methods. However, a few (usually less than five) transformants came up in each plate through the protoplast transformation, and no exconjugants were obtained when *S. lincolnensis* spores were used in conjugation with *E. coli* cells. In this study, by using the mycelia rather than the spores as recipients, with *E. coli* as the donor, we established and optimized an effective method of transferring DNA into *S. lincolnensis* through intergeneric conjugation for the first time.

## 2. Materials and Methods

### 2.1. Strains and Plasmids

*E. coli* DH5α was used as general host for cloning. The methylation-deficient *E. coli* ET12567 (*dam-13::Tn9*, *dcm-6*, *hsdM*, *hsdS*), containing pUZ8002 which is a RK2 derivative, was used as the donor in intergeneric conjugation [18]. Plasmid pIJ773 was used to obtain the apramycin resistance marker *aac(3)IV* and the *oriT* fragment [19]. Plasmid pUWL201 (provided by Professor Huizhan Zhang, East China University of Science and Technology, Shanghai, China), a high-copy-number *Streptomyces* plasmid, was used as the expression vector in streptomycetes [20]. Plasmid pIB139 (provided by Professor Hongyu Ou, Shanghai Jiaotong University, China), carriying ϕC31 *int*/*att* system functions, was used as the integrative expression vector in streptomycetes. *S. lincolnensis* ATCC25466 was reserved in our laboratory.

### 2.2. Media and Culture Conditions

*E. coli* stains were cultured in Luria Bertani (LB) medium [21] at 37 °C, with shaking at 220 rpm, supplemented with appropriate antibiotics as required. The spores of the *S. lincolnensis*, which grew at 30 °C on modified Gauze’s Medium No.1 (MGM, containing 2% soluble starch, 0.5% soybean, 0.1% KNO_3_, 0.05% NaCl, 0.05% MgSO_4_, 0.05% K_2_HPO_4_, 0.001% FeSO_4_, 2% agar, pH 7.0) for 7 days, were scraped and stored in 20% glycerol at −80 °C. Liquid SM medium (containing 1% glucose, 0.4% yeast extract, 0.4% peptone, 0.4% K_2_HPO_4_, 0.2% KH_2_PO_4_, 0.05% MgSO_4_, pH 7.0) [22] was used for *S. lincolnensis* culturing to collect mycelia. 2× YT medium [7] and mannitol soya flour (MS) medium [7] containing 20 mM of MgCl_2_ were used for the intergeneric conjugation in this study. For shake flask fermentation, we used 25 mL of seed medium (containing 2% soluble starch, 1% glucose, 1% soybean, 3% cream corn, 0.15% (NH_4_)_2_SO_4_, 0.4% CaCO_3_, with/without the apramycin) in 250 mL flask at 30 °C with shaking at 220 rpm for 2 days. And 2 mL of the seed cultures was transferred into 25 mL of fermentation medium (10% glucose, 2% soybean, 0.15% cream corn, 0.8% NaNO_3_, 0.5% NaCl, 0.6% (NH_4_)_2_SO_4_, 0.03% K_2_HPO_4_, 0.8% CaCO_3_, with/without the apramycin) and incubated under the same conditions of seed culture but for 7 days.

### 2.3. Conjugation Method

*E. coli* ET12567/pUZ8002/pUWL201apr (or other plasmid containing the *oriT* for conjugation) cells were prepared as previously described [7] with the minor modification. 50 μL of overnight culture was inoculated to 5 mL of LB (containing 25 mg/L kanamycin, 25 mg/L chloramphenicol, 50 mg/L apramycin, and 10 mM MgCl_2_), and incubated at 37 °C with shaking at 220 rpm for 3–4 h to OD_600_ 0.4–0.6. To remove the antibiotics, the cells were collected at 4000 rpm and washed twice with an equal volume of LB (containing 10 mM MgCl_2_) without antibiotics. *E. coli* cells were counted by microscopy, resuspended in 0.5 mL of LB (containing 10 mM MgCl_2_), and used as the donor cells. *S. lincolnensis* mycelia were prepared from 5 mL on day 3 of liquid cultures. The mycelia were collected at 4000 rpm and washed with the equal volume of 10% of glycerol once and 2× YT twice (vortex strongly each time). Finally the mycelia were resuspended in 0.5 mL of 2× YT and used as the recipient.

The prepared *E. coli* cells were transferred into the resuspended mycelia to achieve homogeneity with vortex briefly. The mixture was collected by centrifugation at 4000 rpm, and 400–600 μL of supernatant was removed. The residue was spread on the MS plate (containing 0–50 mM MgCl_2_). The plate was dried in the hoodbench with sterile air, and then incubated overnight in 30 °C with the upside-down. One millilitre of mixed antibiotics (0.5 mg nalidixic acid and 1 mg apramycin) was evenly overlaid on the plate and the surface was dried. The plate was incubated in 30 °C until the exconjugants showed up. The single clones were spread on the new plates containing 25 mg/L of nalidixic acid and 50 mg/L of apramycin to confirm the exconjugants.

### 2.4. PCR Cloning of Lincomycin Resistance Genes and Construction of Over-Expression Vectors

The genomic DNA of *S. lincolnensis* was isolated by Kirby mix procedure [7]. *E. coli* plasmid preparation, restriction digestion, agarose gel electrophoresis and ligation reaction procedures were conducted according to the standard protocols [21]. Streptomycetes plasmids were isolated conducted according to the neutral lysis procedure [7]. The three lincomycin resistance genes were amplified from the genomic DNA by polymerase chain reaction (PCR). Six primers were designed, based on the sequence of *S. lincolnensis* ATCC25466 [5]: rA-F, 5′-GCCCAAGCTTGGAGTCATCTCTCCATGTCT-3′ (with a *Hin*dIII site, underlined) and rA-R, 5′-GCCGGAATTCCTGGAAGTTATCCGGGAGT-3′ (with a *Eco*RI site, underlined) for amplifying *lmrA*; rB-F, 5′-CACCGCTTGAATTCGTTCCATCTGGAGTG-3′ (with a *Eco*RI site, underlined) and rB-R, 5′-GGAAGATCTCCCGGATGTGCTTCTACGA-3′ (with a *Bgl*II site, underlined) for amplifying *lmrB*; rC-F, 5′-GCCGGAATTCCGGAAGATCTCCGACTCCTCCCTGGGAAT-3′ (with a *Eco*RI and a *bgl*II site, underlined) and rC-R, 5′-GCTAGACTAGTCGGCCGATTAACACGCAAGACGCCCTCC-3′ (with a *Spe*I site, underlined) for amplifying *lmrC*. Sequence similarities in databases were searched with the Blast program through the website at EMBL. Clustal W was used for the multiple alignment of amino acid and DNA sequences.

The PCR reaction (50 μL) contained 2.5 U TransStart FastPfu DNA Polymerase (TRANSGEN), 2 mM of MgCl_2_, 10 μL of 5× TransStart FastPfu buffer, 0.5 mM of dNTPs, 100 pM of primer each, 200 ng of template DNA, and 5 μL of DMSO. The PCR onditions were as follows: pre-denaturation for 5 min at 97 °C; denaturation for 30 s at 95 °C, annealing for 30 s at the suitable temperature according to the primers and extension at 72 °C for suitable time according to the length of *lmrA*, *lmrB*, and *lmrC*, repeated for 30 cycles; followed by a final extension for 5 min at 72 °C. The PCR products were purified. DNA sequencing, using the dideoxynucleotide chain termination method with an automated ABI 377A sequencer (Applied Biosystems, USA), was carried out to confirm the correctness of cloned gene.

**Figure 1:**
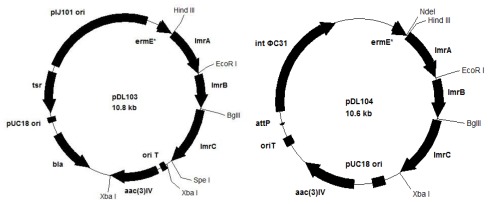
The map of expression vectors pDL103 and pDL104.

The *aac(3)IV* and *oriT* fragment was obtained from pIJ773 after digested with *Xba*I and ligated to pUWL201, yielding pUWL201apr. Genes of *lmrA*, *lmrB*, and *lmrC*, amplified from PCR, were ligated into pUWL201apr one after another by restriction enzyme digestion and ligation reaction procedure, yielding vector pDL103. Using the similar procedure, *lmrA*, *lmrB*, and *lmrC* were ligated into pIB139, resulting vector pDL104 (Figure 1).

### 2.5. Lincomycin Extraction and HPLC Analysis

The fermentation broth was centrifuged at 8000 rpm for 5 min after cultivation, and the supernatant was extracted with 1.5 volumes of methanol. After centrifuged, the extract was freeze-dried for 24 h until no flowed liquid. And the residue was dissolved in methanol to be used.

The concentration of lincomycin was determined by the high pressure liquid chromatography (HPLC) with 18 alkylsilane bonded silica as the stationary phase. 0.05 mol/L borax solution (adjusted pH with 85% phosphoric acid to 6.0): methanol (with the ratio 4:6) was used as the mobile phase. The liquid chromatography was equipped with a 214 nm detector and maintained at a temperature of 30 °C. The flow rate was 0.8 mL per minute. The injection volume was 20 μL.

## 3. Results and Discussion

### 3.1. Optimization of Liquid Media for Recipient Mycelia Growth of *S. lincolnensis*

*E. coli* ET12567/pUZ8002 (containing pUWL201apr) was used as the donor cells in the following studies to determine the optimal condition for intergeneric conjugation.

**Figure 2:**
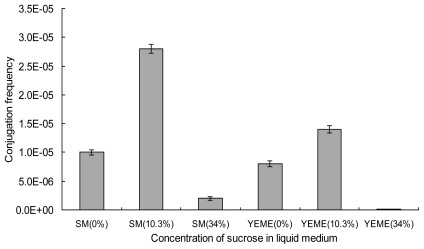
Effects of mycelia growth media on intergeneric conjugation. The competent mycelia were resuspended in 5 mL of 2× YT after being washed with an equal volume of 10% glycerol once and 2× YT twice. 0.5 mL of mycelia suspension and 0.5 mL of approximately 10^7^ of the donor cells were thoroughly mixed and used for the intergeneric conjugation. The conjugation frequency was calculated as the number of exconjugants per input donor cell. Results were the average of three independent experiments. The error bars represent standard deviation.

To select the liquid medium for *S. lincolnensis* mycelia growth, four representative media, SM, YEME, TSB and DNB [7] were tested for their effects on the conjugation efficiency. Exconjugants only appeared when mycelia were collected from liquid SM or YEME. As in the protoplast transformation in other *Streptomyces* [7,23,24], the concentration of sucrose added in the liquid medium was an effective factor for mycelia competence of *S. lincolnensis*. The liquid media added with 10.3% of sucrose led to a significant increase in conjugation frequency (Figure 2). 10.3% of sucrose was favorable for obtaining dispersed *S. lincolnensis* mycelia by loosening cell wall formation, which could have contact with donor cells and absorb plasmids adequately. But high concentration (34%) of sucrose added in both SM and YEME showed adverse results. Mycelia growth was heavily restrained by the high osmotic pressure caused by the high concentration of sucrose. SM medium with 10.3% of sucrose added was optimal for culturing recipient mycelia, compared with YEME, and the conjugation frequency reached 2.8 × 10^−5^, which is almost one fold higher.

### 3.2. Optimization of Recipient Mycelia Growth Conditions

Mycelia age played an important role in competence and transformation. Mycelia incubated for 34 h was suitable for the intergeneric conjugation in *S. peucetius* [16], whereas mycelia during the exponential phase of *Nonomuraea* were optimal [24]. *S. lincolnensis* mycelia of various growing times (2–6 days) were conjugated with *E. coli* cells. According to our study, mycelia of the earlier (1–2 days) or later (5–6 days) stages were not suitable for intergeneric conjugation, with a frequency rate lower than 1.0 × 10^−6^. *S. lincolnensis* mycelia grown for three days at the exponential stage were well dispersed in culture, and this competent stage was favorable for conjugation. The conjugation frequency (7.6 × 10^−5^) was one fold higher than the four-day-old mycelia.

### 3.3. Optimization of Solid Media for Exconjugants Regeneration

**Figure 3:**
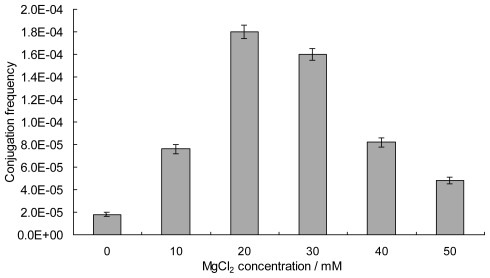
Effects of MgCl_2_ concentration of MS on intergeneric conjugation. Exconjugants were counted after incubation on MS medium (containing 0–50 mM of MgCl_2_) at 30 °C for 7 days. The conjugation frequency was calculated as the number of exconjugants per input donor cell. Results were the average of three independent experiments. The error bars represent standard deviation.

The conjugation medium has a significant influence on the conjugation frequency in streptomycetes [10,18,25–27]. Five applicable solid media, MGM, MS, R2YE [7], ISP Medium 2 (Difco), ISP Medium 4 (Difco), were tested to determine the appropriate medium. Exconjugants were obtained on MS and MGM media, while no exconjugants appeared when using R2YE, ISP2 or ISP4 as the conjugation medium. MS medium, on which the conjugation frequency (7.6 × 10^−5^) increased 3.2-fold compared to the MGM medium (1.8 × 10^−5^), proved to be the most appropriate medium for conjugate regeneration. The conjugation MS medium was further optimized with a supplement of 10, 20, 30, 40, 50 mM of MgCl_2_, respectively. When 20 mM of MgCl_2_ was added to the MS medium, the conjugation frequency of *S. lincolnensis* mycelia increased 9-fold compared without MgCl_2_ (Figure 3). However, the conjugation frequency decreased significantly as the concentration of MgCl_2_ increased, considering the spore formation and mycelia growth were severely inhibited at concentrations of 40 mM or higher for *S. pristinaespiralis* [28]. Thus, MS medium supplemented with 20 mM of MgCl_2_ proved to be optimal for the conjugation of *E. coli*-*S. lincolnensis* mycelia.

### 3.4. Ratio of Donor Cell Numbers to Recipient Mycelia

**Figure 4:**
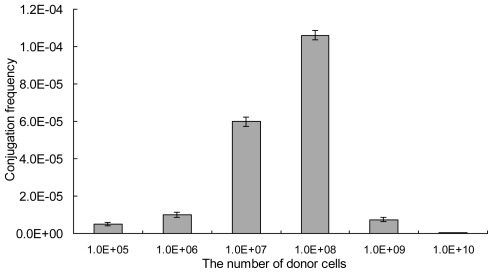
Effects of the number of donor cells on intergeneric conjugation. 0.5 mL of mycelia suspension on the 3rd day and 10^5^–10^10^ of *E. coli* donor cells were used. The conjugation frequency was calculated as the number of exconjugants per input donor cell. Results were the average of three independent experiments. The error bars represent standard deviation.

The ratio of donor cell numbers and recipient mycelia was a crucial parameter in the intergeneric conjugation of streptomycetes [29]. A stationary volume of 0.5 mL of mycelia suspension on third day and a range of *E. coli* donor cells (10^5^–10^10^) were tested. As shown in Figure 4, few exconjugants were obtained when the number of donor cells was less than 10^6^. The conjugation frequency significantly increased with increasing number of donor cells. When the number of the donor cells was up to 10^8^, the conjugation frequency reached the highest frequency of 1.1 × 10^−4^. And this indicated that 10^8^ of donor cells were optimal for the intergeneric conjugation from *E. coli* to *S. lincolnensis* mycelia. Similar results had been observed in the study of actinomycete *Kitasatosporasetae*, where a certain number of donor cells was required to achieve the highest frequency [25].

### 3.5. Over-Expression of *lmrABC* in *S. lincolnensis*

Further studies were carried out in order to practice the reliability of this intergeneric conjugation. The multiple copy vector pDL103 and the integrative vector pDL104 expressing *lmrABC* driven by the promoter ermE* were constructed. These two types of expression vectors were subsequently transformed into *S. lincolnensis* through the intergeneric conjugation as described above. In the three separate conjugation experiments, the average conjugation frequencies were 1.2 × 10^−4^ for pDL103 and 9.6 × 10^−5^ pDL104, respectively. The multiple copy and integrative vectors affected the intergeneric *E. coli*-mycelia conjugation least.

The resulting exconjugate strain, over-expression of *lmrABC* in *S. lincolnensis*, named D201 (containing pDL103) and D202 (integrated with pDL104), were easily screened and selected with apramycin and nalidixic acid after conjugation. For growth status, exconjugants of *S. lincolnensis* carrying pDL103 or pDL104 did not differ from the parent strain in the growth rate and colony size. The culture of the flask fermentation was extracted and the lincomycin titer was measured by HPLC. The production of lincomycin in fermentation broth of strains D201 and D202 were 94.8 ± 3.6 mg/L and 85.7 ± 3.4 mg/L, with a ratio of 52.9% and 38.3% higher than the parent strain (62.0 ± 2.9 mg/L), respectively.

## 4. Conclusions

In conclusion, a simple and efficient intergeneric conjugation method of transferring pasmid DNA from *E. coli* to *S. lincolnensis* using mycelia was developed and optimized for the first time. Recently, a few intergeneric conjugation methods using mycelia for plasmid transferring have been reported in poorly sporulating *S. fradiae* [14] and *S. peucetius* [16], and in reluctant PEG-mediated protoplast transformation of *S. rimosus* [15]. Without preparation of protoplasts or spores, the use of mycelia streamlines conjugation with *Escherichia coli*. The *E. coli*-mycelia conjugation method from our results combined with those reports [13–17], could be an attractive biotechnology for introducing foreign DNA to mycelia organisms, especially for refractory streptomycetes, on account of its timesaving, convenience, and high transformation frequency advantages.
